# Curing Behavior of Sucrose with *p*-Toluenesulfonic Acid

**DOI:** 10.3390/polym15234592

**Published:** 2023-11-30

**Authors:** Shunsuke Sakai, Shuoye Chen, Miyuki Matsuo-Ueda, Kenji Umemura

**Affiliations:** Laboratory of Sustainable Materials, Research Institute for Sustainable Humanosphere, Kyoto University, Gokasho, Uji, Kyoto 611-0011, Japan; sakai.shunsuke.46r@st.kyoto-u.ac.jp (S.S.); chen.shuoye.4z@kyoto-u.ac.jp (S.C.); matsuo.miyuki.4d@kyoto-u.ac.jp (M.M.-U.)

**Keywords:** bio-based adhesive, curing behavior, sucrose, *p*-toluenesulfonic acid

## Abstract

With respect to the fossil resources shortage, the development of bio-based wood adhesives is an important research topic in wood science. There has been research on using sucrose for bio-based adhesives. However, a high acid catalyst content and a high hot-pressing temperature are required when manufacturing particleboards. In this study, to explore the possibility of *p*-toluenesulfonic acid (PTSA) as a promising acid catalyst for sucrose-based adhesives, the curing behavior of sucrose with PTSA (Suc-PTSA) was clarified. The thermal analysis results showed that the thermal properties of sucrose decreased significantly with the addition of PTSA. Based on the results of the insoluble matter rate, the optimal mixture ratio and heating conditions were determined to be 95:5 and 180 °C for 10 min, respectively. According to the results of FT−IR, the heat-treated Suc-PTSA contained furan compounds. In the context of the dynamic viscoelasticity, the onset temperature at which the storage modulus (*E′*) begins to rise was significantly lower than those of the other sucrose-based adhesives. PTSA has the potential to cure sucrose more efficiently and at lower temperatures than previous sucrose-based adhesives, making it a promising acid catalyst for sucrose.

## 1. Introduction

In recent years, environmental and resource problems have become more serious globally [1]. With respect to fossil resource shortages, the utilization of bioresources has attracted significant attention [2]. In addition to the research on biomass energy, the research on the development of materials derived from bio-resources is also an important issue [3,4]. Generally, wood-based materials are representative biomaterials. According to the United Nations Food and Agriculture Organization statistics [5], the demand for wood-based materials, especially wood-based panels, such as particleboards, is expected to increase significantly. However, synthetic resin adhesives need to be applied to the raw wood materials during the manufacturing process. The raw materials used for synthetic resin adhesives are chemical compounds derived from fossil resources. Moreover, some adhesives contain formaldehyde, which is known to pose health risks [6]. Considering the breakaway from dependence on fossil resources, synthetic resin adhesives should be replaced as much as possible with bio-based adhesives derived from bio-resources as much as possible [7,8]. Various bio-based wood adhesives have been proposed, including protein [9], starch [10], and aromatic-based adhesives [11]. However, many bio-based wood adhesives that have been studied require complicated chemical modification [12]. Therefore, the development of bio-based adhesives with simple preparation is desired.

In the last decade, there has been research on using sucrose for bio-based wood adhesives [13,14]. Sucrose is a well-known bioresource that is safe and inexpensive [15,16]. When sucrose is used as an adhesive, acidic compounds such as citric acid (CA) and ammonium dihydrogen phosphate (ADP) are required [14,17]. In these studies, particleboards were manufactured by applying an aqueous solution of sucrose with CA or ADP to the wood particles, and then hot-pressing them. The particleboards used in these studies exhibited good mechanical properties and water resistance. The effective hot-pressing temperature, time, and weight ratio were 200 °C, 10 min, and 85:15, respectively. The principal curing mechanism of sucrose with an acid compound involves the formation of furan polymers through the formation of 5-hydroxymethylfurfural (HMF). However, a high hot-pressing temperature and high ADP content are required when manufacturing particleboards.

HMF is a promising chemical compound [18], and various methods to produce it have been researched [19,20]. Notably, *p*-toluenesulfonic acid (PTSA) was identified as an effective catalyst for enhancing HMF yield in a water medium [21,22]. PTSA appears to be an effective catalyst for bio-based adhesives using sucrose; however, its feasibility has not yet been investigated. In this study, we investigated the curing behavior of sucrose with PTSA (Suc-PTSA) and aimed to clarify whether PTSA cures sucrose at lower temperatures and with reduced acid addition compared to previously reported sucrose-based adhesives.

## 2. Materials and Methods

### 2.1. Materials

Sucrose, *p*-toluenesulfonic acid monohydrate, ammonium dihydrogen phosphate, and anhydrous citric acid of all guaranteed reagent grades were purchased from Nacalai Tesque, Inc. (Kyoto, Japan), and used without further purification.

### 2.2. Sample Preparation

Sucrose and PTSA were dissolved in distilled water in five weight ratios: 100:0, 98:2, 95:5, 90:10, and 85:15. The concentration of the solution was adjusted to 50 wt% by considering the hydrate content of the PTSA. The pH of each solution was measured using a pH meter D-71 (Horiba Ltd., Kyoto, Japan). Two g of each solution were poured into an aluminum cup and dried at 80 °C for 12 h in an oven. The dried mixtures were heated at 120, 140, 160, 180, and 200 °C for 10 min. The heated mixtures were vacuum-dried at 60 °C for 15 h and pulverized into particles small enough to fit through a 60-mesh filter. The preparation conditions of the samples are shown in Table 1.

Based on the previous studies [13,14], aqueous solutions of sucrose and ADP (Suc-ADP) and sucrose and CA (Suc-CA) were prepared for dynamic viscoelastic analysis. The weight ratios of the Suc-ADP and Suc-CA were 90:10 and 75:25, respectively. The concentration of each solution was adjusted to 50 wt%.

### 2.3. Thermal Analysis

The dried mixtures were further vacuum-dried at 60 °C for 15 h and pulverized into particles small enough to fit through a 60-mesh filter. Differential scanning calorimetry (DSC) and thermogravimetric analysis (TGA) were performed using a DSC 25 (TA Instruments Japan Inc., Tokyo, Japan) and TGA 55 (TA Instruments Japan Inc.), respectively. For the DSC, a small hole was made in the cover of the aluminum pan using tweezers. The samples were scanned from room temperature to 400 °C at the rate of 10 °C/min under N_2_ purging.

### 2.4. Measurement of Insoluble Matter Rate

First, 3 g of each heated mixture was boiled in 500 mL boiling water for 4 h. After boiling, the solution was filtered through filter paper, and the insoluble matter remaining on the paper was vacuum-dried at 60 °C for 15 h. The rate of insoluble matter in the heated mixture was determined using the following equation:

(1) $$\mathrm{Insoluble}\;\mathrm{matter}\;\mathrm{rate}\;(\%)=\frac{W_{a}}{W_{b}}\times 100$$

where *W_a_* is the dry weight of the insoluble matter after boiling water immersion, and *W_b_* is the dry weight of the heated mixture before boiling. The pH of the filtrate was measured at 20 °C using a D-71 pH meter. The experiments were performed three times, and the average values were calculated.

### 2.5. FT−IR Analysis

Each of the dried mixture, heated mixture, and insoluble matter were vacuum-dried at 60 °C for 15 h and ground into a powder. FT−IR spectra were obtained using an infrared spectrophotometer (FT/IR-4200 JASCO Corporation, Tokyo, Japan) using the KBr disk method and recorded at 4000–400 cm^−1^ wavenumbers with 32 scans at 4 cm^−1^ resolution.

### 2.6. Dynamic Viscoelastic Analysis

The method of Follensbee et al. [23] was adopted to measure the dynamic viscoelasticity. A glass fiber filter GA-200 (ADVANTEC, Tokyo, Japan) was cut to obtain a strip (measuring 40 mm × 3 mm × 0.8 mm and weighing about 0.02 g), which was then oven-dried. The strip was dipped into the solution. The excess solution was removed so that the weight of the strip with the solution was approximately 0.2 g. The dynamic viscoelasticity was measured using a dynamic viscoelastic spectrometer DVA-200 (IT Keisoku Seigyo Co., Ltd., Osaka, Japan) by scanning from room temperature to 250 °C at a rate of 10 °C/min at 10 Hz.

## 3. Results and Discussion

### 3.1. Thermal Properties of the Dried Mixtures of Suc-PTSA

**Figure 1:**
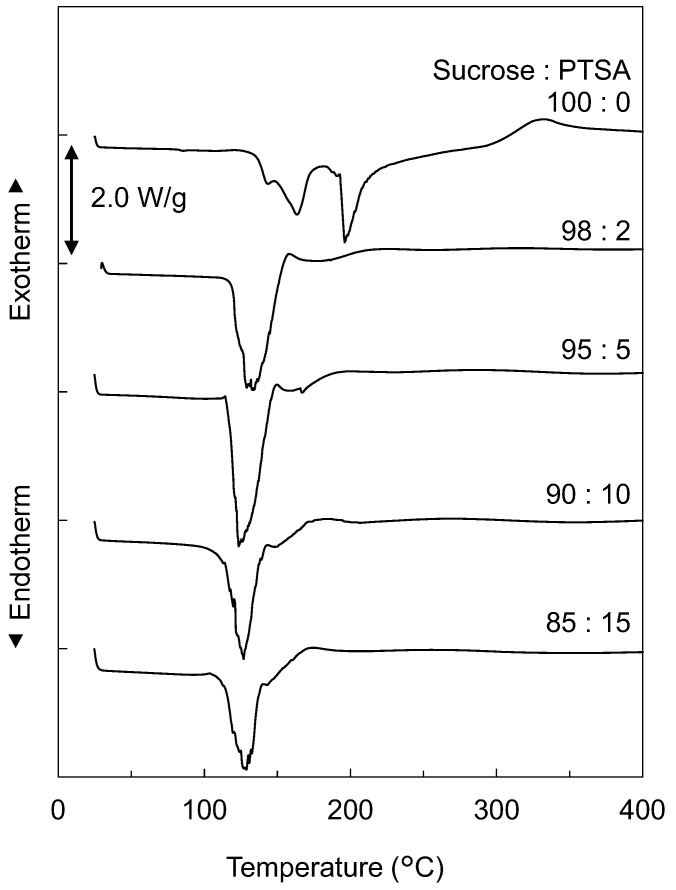
DSC curves of the dried mixtures of sucrose and *p*-toluenesulfonic acid (Suc-PTSA) with different mixture ratios.

**Figure 2:**
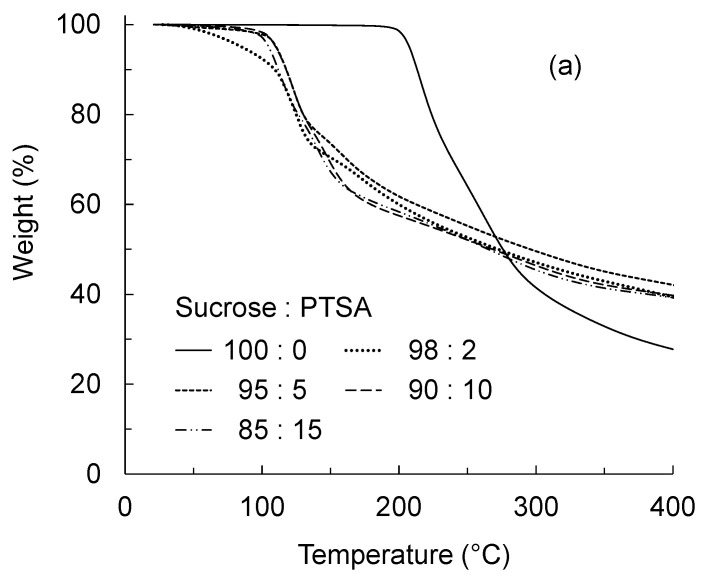

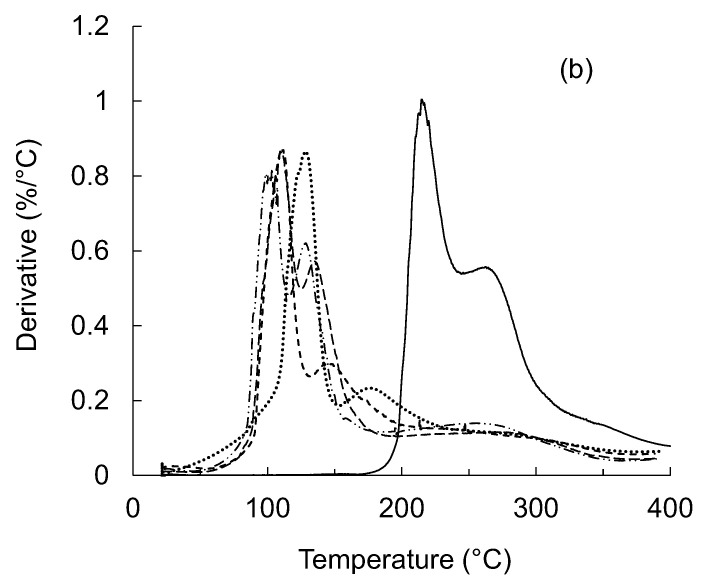
(**a**) TG curves; and (**b**) DTG curves of the dried mixtures of sucrose and *p*-toluenesulfonic acid (Suc-PTSA) at different mixing ratios.

To investigate the thermal behavior of Suc-PTSA, DSC and TGA measurements were performed on the dried mixtures. Figure 1 shows the DSC curves of the dried mixtures of Suc-PTSA, and Figure 2 shows the (a) TG and (b) derivative TG (DTG) curves. In the case of 100:0 (sucrose only), endothermic peaks were observed at approximately 145, 165, and 200 °C in the DSC curve. The TG curve showed that the weight loss started at approximately 200 °C. In the DTG curves, two peaks at are observed approximately 200 and 270 °C. The endothermic peaks at around 145 and 165 °C were not accompanied by weight loss, and this lack of weight loss was attributed to the melting of sucrose [24,25]. On the other hand, the endothermic peak at approximately 200 °C was accompanied by weight loss, suggesting that the loss was due to the thermal decomposition of sucrose, i.e., caramelization [26]. The peak observed at approximately 270 °C in the DTG was thought to be due to the carbonization of sucrose [27].

An entirely different behavior was observed when PTSA was added. The thermal behavior was almost the same for all mixture ratios. The DSC curves showed an endothermic peak at approximately 120–130 °C, and no thermal behavior was observed thereafter. In the TGA curves, the weight decreased rapidly from 100 °C to 160 °C. According to the DTG curves, marked weight loss was observed at 120–130 °C. After proceeding at a rapid pace, the weight loss slowed, but continued up to 400 °C. In the case of sucrose only, the endothermic peak and weight loss were observed at approximately 200 °C, but in a significantly lower temperature range of 120–130 °C in Suc-PTSA. This indicates that, with the addition of PTSA, the reaction temperature of sucrose with heat decreased. While three endothermic peaks were observed in sucrose only, only one endothermic peak, at 120–130 °C, was observed in Suc-PTSA. This means that when PTSA was added to sucrose and subsequently heated, the endothermic reaction and weight loss of sucrose occurred simultaneously.

Umemura et al. [13] clarified the thermal properties of a dried mixture of Suc-ADP. They reported that the thermal properties of Suc-ADP were lower than those of sucrose. The endothermic reaction and weight reduction exhibited peaks in the range of 140–154 °C. In the present study, the thermal behavior of the Suc-PTSA was similar to that of Suc-ADP, with temperatures up to 20 °C lower than that of Suc-ADP. It seems to be that PTSA was able to reduce the thermal properties of sucrose at lower temperatures than ADP.

### 3.2. Insoluble Matter Rates of the Heated Mixtures against Boiling Water

**Figure 3:**
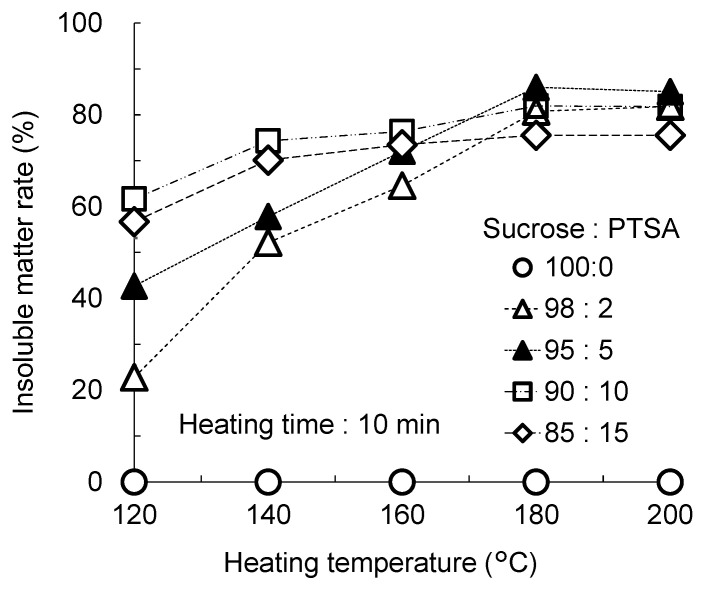
Effects of heating temperature on the insoluble matter rate of Suc-PTSA with different mixture ratios.

To evaluate the degree of curing, the rates of insoluble matter of heated mixtures were calculated. The results are shown in Figure 3. In the case of 100:0 (sucrose only), the rate of insoluble matter remained at 0%, irrespective of heating temperature. This means that the heated sucrose is soluble in boiling water. With the addition of PTSA, the heated mixtures showed insolubility at all mixture ratios and at all heating temperatures. This indicated that the addition of PTSA and heating resulted in the formation of some polymers insoluble in boiling water. In the cases of 98:2 and 95:5 ratios, the insoluble matter rates at 120 °C were low (23% and 43%, respectively). From 120 °C to 180 °C, the rate increased linearly, and there was no difference between 180 °C and 200 °C. When heated above 180 °C, the insoluble matter rate was over 80%. In the cases of 90:10 and 85:15 ratios, insoluble matter rates of over 58% were observed, even at 120 °C. When higher amounts of PTSA are used, the reaction leads to the formation of insoluble matter occurred at lower temperatures. This suggests that PTSA promotes the conversion of sucrose into insoluble matter.

In this study, the highest insoluble matter rate, (86%) was obtained at a mixture ratio of 95:5 and a heating temperature of 180 °C. It was confirmed that Suc-PTSA was sufficiently cured under the heating conditions of 180 °C for 10 min.

Table 2 shows the pH values of the filtrate after the boiling treatment. The 100:0 solution was neutral, while the solution in the cases of Suc-PTSA was acidic. The pH value ranged from 3.06 to 2.40; the pH value decreased as the amount of PTSA increased. It seems that PTSA dissolved during the boiling treatment. Assuming that PTSA was not involved in the curing reaction and acted as a catalyst, it was expected that the insoluble matter was derived from only sucrose. Accordingly, the insoluble matter rate by the sucrose ratio was calculated by dividing the insoluble matter rate based on the sucrose content of the Suc-PTSA. As a result, the overall trend remained the same, but the values increased substantially for mixtures with more PTSA. Approximately 90% of the insoluble matter rates based on the sucrose content was obtained under all conditions except for 98:2 heated at 180 °C. In other words, about 90% of the sucrose was converted to a substance that was insoluble in boiling water.

In a previous study [13], a similar experiment was performed using Suc-ADP. For all mixture ratios of sucrose and ADP, the insoluble matter rate increased with increasing heating temperature up to 200 °C. The effective mixture ratio was 90:10, and when heated at 180 °C, the insoluble matter rate was 82%. For Suc-PTSA and Suc-ADP, both at a mixture ratio of 95:5 and heated at 180 °C, the insoluble matter rates were 90% and 77%, respectively. At 160 °C, the rates were 76% and 48% for Suc-PTSA and Suc-ADP respectively. This suggests that PTSA is capable of curing sucrose at lower heating temperatures, with a lower quantity required than ADP.

### 3.3. Chemical Changes and Expected Curing Reaction System of Suc-PTSA

**Figure 4:**
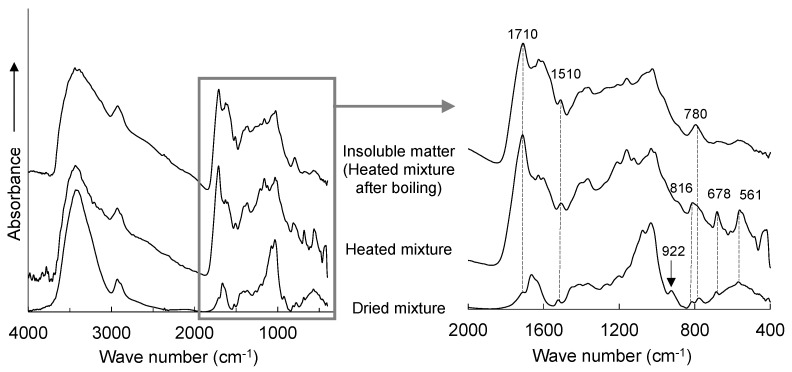
FT−IR spectra of each Suc-PTSA mixture at a ratio of 95:5.

**Figure 5:**
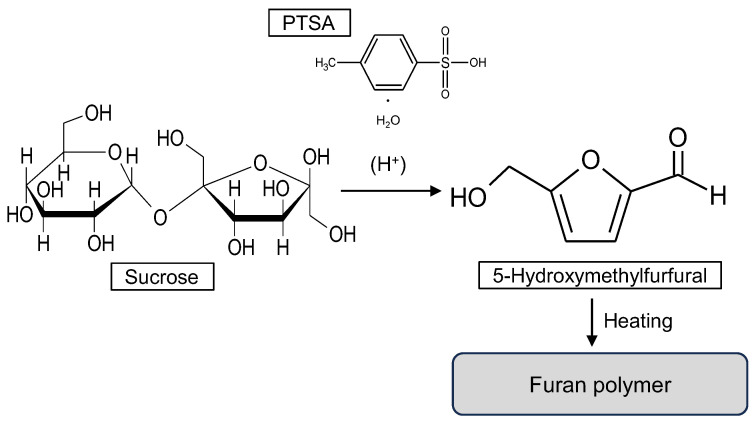
Expected reaction system for Suc-PTSA.

FT−IR spectra were obtained to investigate the chemical changes in Suc-PTSA after drying, heating, and boiling treatments. Figure 4 shows the infrared spectra of the dried mixture, the mixture heated at 180 °C for 10 min, and insoluble matter (heated mixture after boiling) at a mixture ratio of 95:5. Several characteristic peaks were observed between 2000–400 cm^−1^. In the dried mixture, a small peak of infrared absorption was observed at 922 cm^−1^, which was attributed to the C-C stretching vibration of the pyranose ring derived from sucrose [28,29]. This peak almost disappeared in the heated mixture. This indicated that heating degraded sucrose. The peaks at 1710, 1510, 816, 780, 678, and 561 cm^−1^ were more clearly observed in the heated mixture. The peak at 1710 cm^−1^ was ascribed to the C=O bond derived from the carbonyl group [30,31], while the peaks at 1510 cm^−1^ and 780 cm^−1^ corresponded to the stretching vibration of C=C and CH=CH bonds in the furan ring, respectively [32,33]. These results indicate that furan compounds and carbonyl groups were formed due to the dehydration-condensation of the decomposed products. According to a previous study on the curing reaction process of sucrose-based adhesives, acid degradation products such as HMF are raw materials that undergo complex reactions to form polymers [34]. Furthermore, HMFs are known to undergo self-condensation in acidic catalysts [35]. Based on these reports [28,29,30,31,32,33,34,35], the expected reaction system of Suc-PTSA is shown in Figure 5. A similar reaction would have occurred in the present study: heating the polymerized acid degradation products containing HMF from sucrose. In the insoluble matter after boiling treatment, the peaks at 816, 678, and 561 cm^−1^ disappeared, while other peaks, such as those at 1710, 1510, and 780 cm^−1^, were observed. The peaks at 816 and 678 cm^−1^ were attributed to the C-H and C-C-C out-of-plane angular vibrations of the benzene ring, respectively, and the peak at 561 cm^−1^ was attributed to the SO_3_ angular vibration of the sulfo group, which were derived from PTSA [36,37]. Considering the results of the insoluble matter rate, the heated mixture would be a polymer containing mainly furan compounds. Since all PTSA-derived peaks were not observed in the spectra of insoluble matter, PTSA was not involved in the curing reaction. This means that in the measurement of the insoluble matter rate, the higher the amount of PTSA added, the lower the pH of the filtrate after the boiling treatment. It was found that PTSA would act as an acidic catalyst.

### 3.4. Dynamic Viscoelasticity of Suc-PTSA

**Figure 6:**
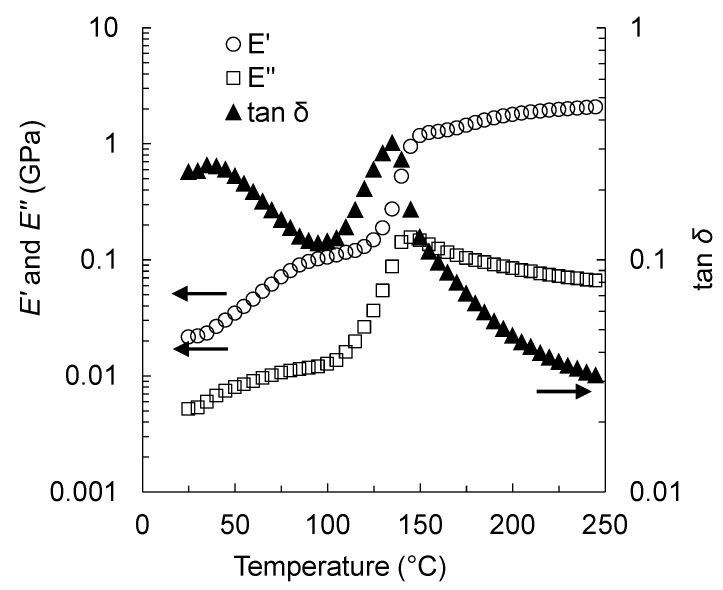
Changes in dynamic viscoelastic properties during the curing process of Suc-PTSA solution.

The dynamic viscoelastic properties of an aqueous Suc-PTSA were measured, and the results are shown in Figure 6. The storage modulus (*E′*) increased slowly up to 120 °C, sharply increased to 145 °C, and then continued to increase slowly until the end of the measurement. The loss modulus (*E″*) increased rapidly from 110 to 140 °C and decreased thereafter. The Tan *δ* peaked at 135 °C, followed by a significant decrease. The behaviors of *E′* and tan *δ* were found to be similar to those of the aqueous phenol-formaldehyde (PF) resin. Umemura et al. [38] used a similar method to measure the curing process of a commercial aqueous PF resole resin, in which the value of *E′* increased gradually up to 114 °C, then increased dramatically to 135 °C and soon reached a constant value. The values of *E′* and tan *δ* exhibited peaks at 129 and 123 °C, respectively. The authors analyzed the behavior as follows: from 20 to 114 °C, *E′* increased slowly as the curing reaction occurred in the presence of water. From 114 to 135 °C, gelation of the resin occurred, the structure changed to glassy, and the value of *E′* remained almost constant thereafter. Kim et al. [39] regarded the peak of tan *δ* of PF resin as the phase transition point during the curing process. Based on the above, we can describe the curing process of Suc-PTSA as follows:

The increase in *E′* and *E″* from room temperature to 110 °C can be attributed to the evaporation of water. As shown in Figure 1 and Figure 2, the dried Suc-PTSA mixture did not react with the heat from room temperature to 100 °C. The samples used in this measurement were glass fiber filters impregnated with an aqueous solution. Therefore, the evaporation of water affects the viscoelasticity. The marked changes in *E′*, *E″*, and tan *δ* from 110 to 145 °C were due to the curing reaction with dehydration-condensation. At around 145 °C, the curing of Suc-PTSA was almost completed, and the increase in *E′* became gradual. At this time, Suc-PTSA became almost glassy and formed a polymer. However, a slight increase in *E′* continued, suggesting that the curing was incomplete. This means that, in the results of the insoluble matter rate, heating above 180 °C was necessary to reach sufficient curing.

**Figure 7:**
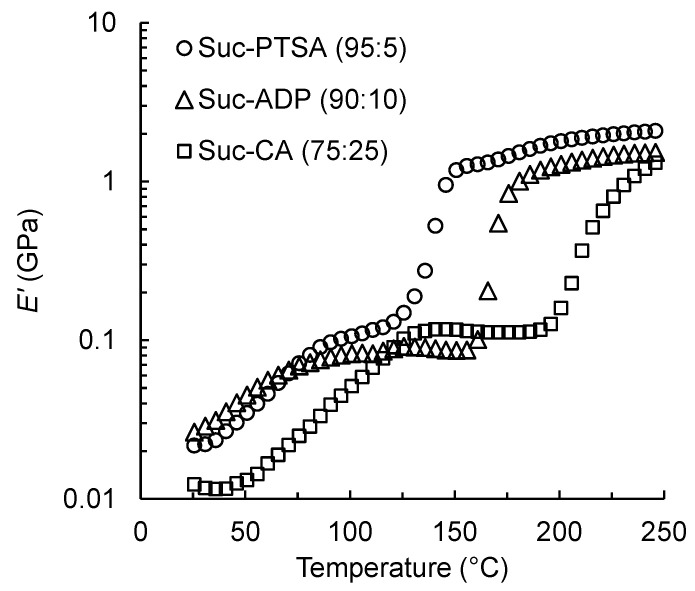
Storage modulus (*E′*) of the three sucrose-based adhesives.

Here, the *E′* of Suc-PTSA was compared with that of the conventional sucrose-based adhesives Suc-ADP and Suc-CA. The mixture ratios of Suc-ADP and Suc-CA were 90:10 and 75:25, respectively, which were previously identified as the optimal ratios [13,14]. The results are shown in Figure 7. A clear difference appeared in the onset temperatures at which *E′* began to increase significantly. *E′* of Suc-ADP increased rapidly from 160 °C to 180 °C. Zhao et al. [17] examined the effect of hot-pressing temperature on the mechanical properties of particleboards bonded with Suc-ADP. When the hot-pressing temperature was in the range of 140–200 °C and the time was 10 min, the boards exhibited positive correlations between mechanical properties and hot-pressing temperatures. The maximum property values were obtained at 200 °C for 10 min. Suc-CA showed the highest onset temperature at which *E′* began to increase. It increased from 190 to 250 °C, which was the highest measurement temperature. Umemura et al. [40] also examined the effect of the hot-pressing temperature on the mechanical properties of the particleboards bonded with Suc-CA. They concluded that the optimal hot-pressing condition was 200 °C for 10 min. For Suc-PTSA, *E′* increased at a significantly lower temperature than those of the two sucrose-based adhesives. Based on the results of dynamic viscoelasticity, Suc-PTSA aqueous solution can be used as an adhesive for wood-based materials. In addition, there seems to be a possibility of reducing the hot-pressing temperatures or time in the production of wood-based materials. In addition, PTSA required less acid addition to sucrose. Therefore, PTSA could be expected to serve as a promising acid catalyst to be added to sucrose.

## 4. Conclusions

In this study, the possibility of using PTSA as an acid catalyst in sucrose-based adhesives was investigated. The thermal properties, insoluble matter rate of the heated mixtures, and chemical changes in Suc-PTSA were evaluated. The dynamic viscoelasticity of Suc-PTSA was compared with that of conventional sucrose-based adhesives. The results are summarized below:

1. The thermal properties of Suc-PTSA were significantly lower than those of sucrose only. Suc-PTSA exhibited an endothermic peak and marked weight loss at 120–130 °C.
2. Based on the results of the insoluble matter rate, the effective mixture ratio and heating conditions were 95:5 and 180 °C for 10 min. Then, 90% of the sucrose was changed into an insoluble substance in boiling water.
3. With the addition of PTSA and heating, sucrose was almost completely decomposed. Sucrose was converted to a highly water-resistant substance through the formation of furan rings and carbonyl groups.
4. The *E′* of the Suc-PTSA solution began to increase from 110 °C to 145 °C, and the curing proceeded moderately. Compared to Suc-ADP and Suc-CA, Suc-PTSA was found to increase *E′* at lower temperatures.

Consequently, it was clarified that PTSA cured sucrose at lower temperatures and with reduced acid addition compared to conventional sucrose-based adhesives. Therefore, Suc-PTSA is expected to serve as a promising bio-based adhesive and has the potential to reduce the manufacturing energy when applied to wood-based materials. In future research, we would like to investigate the bonding properties and mechanisms of underlying wood-based materials manufactured using Suc-PTSA.

## Figures and Tables

**Table 1:** Preparation of samples.

Mixture Ratios Sucrose: *p*-Toluene Sulfonic Acid (PTSA)	pH (50 wt%)	Drying		Heating	
		Temperature (°C)	Time (h)	Temperature (°C)	Time (min)
100:0	5.79	80	12	120, 140, 160, 180, 200	10
98:2	0.84				
95:5	0.54				
90:10	0.29				
85:15	0.18				

**Table 2:** pH value of the filtrate at 20 °C after insoluble matter rate measurement.

	Sucrose:PTSA				
	100:0	98:2	95:5	90:10	85:15
Average	7.24	3.06	2.90	2.53	2.40
SD (*n* = 5)	0.04	0.07	0.06	0.05	0.07

## Data Availability

The data presented in this study are available upon request from the corresponding author.
